# Physical activity and its impact on cardiovascular health in pediatric kidney transplant recipients

**DOI:** 10.1007/s00467-023-06248-7

**Published:** 2023-12-16

**Authors:** Lena Kohlmeier, Jeannine von der Born, Elena Lehmann, Kerstin Fröde, Carl Grabitz, Anne-Sophie Greiner, Alexander A. Albrecht, Nima Memaran, Rizky I. Sugianto, Uwe Tegtbur, Bernhard M. W. Schmidt, Nele Kanzelmeyer, Anette Melk

**Affiliations:** 1 Department of Pediatric Kidney, Liver and Metabolic Diseases, Hannover Medical School, Carl-Neuberg-Str. 1, 30625 Hannover, Germany; 2 Institute of Sports Medicine, Hannover Medical School, Hannover, Germany; 3 Department of Nephrology and Hypertension, Hannover Medical School, Hannover, Germany

**Keywords:** Children, Kidney transplantation, Physical activity, Blood pressure, Metabolic syndrome, Diastolic function

## Abstract

**Background:**

Cardiovascular (CV) morbidity after kidney transplantation (KTx) in childhood is of increasing importance. In light of a high prevalence of CV risk factors, protective measures such as physical activity (PA) come into focus. Our aim was to comprehensively assess PA in pediatric KTx recipients and evaluate its impact on CV health.

**Methods:**

Forty-eight patients were assessed for frequency, duration, intensity, and setting of PA using the “Motorik–Modul” PA questionnaire. Walking-based activity was measured by accelerometer in a subgroup (*n* = 23). CV risk factors and subclinical CV organ damage were determined. The impact of PA on CV parameters was analyzed using linear regression models.

**Results:**

Fifty-two percent of pediatric KTx recipients did not reach WHO recommended PA level; 54% did not engage in PA with vigorous intensity (VPA). Twenty-nine percent indicated an extremely inactive lifestyle (< 120 min/week of moderate to vigorous intensity PA, MVPA). Compared to the healthy German KiGGS cohort, KTx recipients specifically lacked engagement in sport activities (KTx: 129 min/week; 95%CI, 97–162 vs. KiGGS, 242 min/week; 95%CI, 230–253). VPA was associated with lower systolic blood pressure (*p* = 0.024) and resting heart rate (*p* = 0.005), MVPA with fewer components of the post-transplant metabolic syndrome (*p* = 0.037), and better left ventricular diastolic function (*p* = 0.006).

**Conclusions:**

A considerable lack of PA, especially VPA, exists in young KTx recipients. PA was positively associated with important parameters of CV health. While long-term CV protection through PA seems promising in pediatric KTx recipients, specific educational approaches are most likely needed to increase patients’ engagement in sport activities.

**Graphical abstract:**

**Figure:**
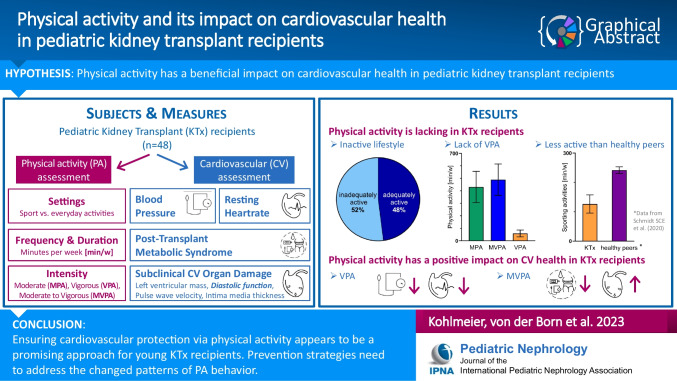
A higher resolution version of the Graphical abstract is available as Supplementary information

**Supplementary Information:**

The online version contains supplementary material available at 10.1007/s00467-023-06248-7.

## Introduction

Children and adolescents with chronic kidney disease (CKD) and subsequent kidney transplantation (KTx) suffer from high cardiovascular (CV) morbidity [1–3] as a result of the accumulation of classical and non-classical risk factors during the course of disease progression [4, 5]. After KTx, this is exemplified by a high prevalence of post-transplant metabolic syndrome (PTMS), which is 16-fold higher in pediatric KTx recipients than in healthy peers [6]. Early vascular changes are frequently detected in pediatric KTx recipients by increases in aortic pulse wave velocity (PWV) and carotid intima media thickness (IMT) indicating arterio- and atherosclerosis [1]. Cardiac alterations such as left ventricular hypertrophy (LVH) and diastolic dysfunction [7] have been described in this high-risk group. It is therefore not surprising that CV complications are among the most common causes of death after KTx in childhood and a major limitation for long-term survival [8].

A sedentary lifestyle has been identified as a CV risk factor for children and adolescents with chronic conditions [9, 10]. In children with CKD or after KTx, limited data suggests a low level of physical activity (PA) combined with a decreased exercise capacity [11] and muscle weakness [12]. The positive effect of PA on classical CV risk factors has been extensively described [13, 14]. In fact, adult KTx recipients engaging in more PA presented with a better preservation of their kidney function and decreased CV and all-cause mortality [15]. The current recommendations of the World Health Organization (WHO) for healthy children and adolescents suggest an average of 60 min of daily moderate to vigorous intensity physical activity (MVPA) including PA with vigorous intensity (VPA) for at least 3 days a week in order to gain most health benefits [16]. Whether these suggestions also apply to the same extent to children and adolescents with chronic conditions, including children with CKD and KTx, remains elusive.

As there is limited data on PA for children after KTx, the aim of this study was to comprehensively assess PA in pediatric KTx recipients. The intention was to capture not only PA’s frequency and duration but also the intensity and the setting in which PA was performed. Finally, we meant to analyze PA’s potential impact on CV risk factors and subclinical CV target organ damage.

## Methods

### Study design and population

In this cross-sectional study, we investigated 48 children, adolescents and young adults, who underwent KTx before the age of 18 years, from Hannover Medical School’s outpatient clinic. Patients were recruited between November 2020 and June 2021. We only included patients with a functioning graft. One patient had to be excluded from echocardiographic measurements because of congenital heart disease. A further exclusion criterion was an age below 5 years because of missing reference values for some of the CV measures. All patients and caregivers gave informed consent before participation. The study was approved by the institutional review board (No. 504) and fully complies with the Declaration of Helsinki.

### Basic patient characteristics

Basic patient characteristics regarding underlying disease, transplantation details, and dialysis prior to transplantation and medication were obtained from patients’ files. Weight, height, and waist circumference were measured. Body mass index (BMI) with *z*-score (BMIz) as well as *z*-scores for weight, height, and waist circumference (WCz) was calculated [17, 18]. Overweight was defined as BMIz > 1.036 and ≤ 1.645 (85th to 95th percentile (pct.)) and obesity as BMIz > 1.645 (95th pct.) [19]. Blood and urine samples were collected and analyzed at one central laboratory (SYNLAB, Heidelberg, Germany). The following parameters were measured: serum creatinine, low density lipoprotein (LDL), high density lipoprotein (HDL), total cholesterol, triglycerides, urine creatinine, and urine albumin. Estimated glomerular filtration rate (eGFR) was calculated using the Schwartz formula [0.41 × height (cm)/plasma creatinine (mg/dl)] [20].

### Physical activity assessment

PA was assessed by the “Motorik–Modul” PA questionnaire (MoMo-PAQ) from the MoMo study, which is part of the German Health Interview and Examination Survey for Children and Adolescents (*Studie zur Gesundheit von Kindern und Jugendlichen in Deutschland,* KiGGS) [21]. This allowed comparing our study population to a representative cohort of German children and adolescents recruited for the KiGGS study.

The MoMo-PAQ shows comparable reliability and validity to international PA questionnaires for children and adolescents [22]. It gathers information about self-reported habitual PA in different settings: sports at school, sports in clubs, unorganized sports, and activities of everyday life. The items measure frequency, duration, and intensity of PA during a regular week. The intensity of each activity was converted into the metabolic equivalent of task (MET) on the basis of the classifications of Ainsworth et al. [23]. MET values (ratio of work metabolic rate to a standard resting metabolic rate (RMR) of 1.0 kcal·kg^−1^·h^−1^) describe the energy consumption for specific physical activities. One MET is the estimated RMR or the energy consumption of a person at rest. MET values between 3 and 6 indicate moderate PA (MPA), and MET values > 6 described PA with vigorous intensity (VPA), respectively. Based on this classification, we calculated minutes per week spent in PA of moderate (MPA) or vigorous intensity (VPA) as well as the combination thereof, designated as moderate to vigorous intensity PA (MVPA; MET values ≥ 3). To objectively measure walking-based activity, a subgroup of patients (*n* = 23), who agreed to be equipped with an accelerometer (Forerunner 35, Garmin, Schaffhausen, Switzerland) for 7 days to calculate mean steps per day, was analyzed. The remaining 25 KTx recipients refused the accelerometer measurement.

### Cardiovascular assessment

*Standardized blood pressure* (BP) measurements were performed with a validated oscillometric device (Dinamap v100, GE Healthcare, Chicago, IL) after 5 min of rest in a seated position, one measurement on the left and one at the right arm. Values derived at the right arm [24] were expressed as *z*-scores normalized for sex, age, and height for systolic (SBPz) and diastolic BP (DBPz) [25]. Arterial hypertension was defined as either SBPz and/or DBPz > 1.65 (> 95th pct.) or antihypertensive medication use [26] based on the information collected from patients’ clinical file. The device was also used to measure resting heart rate (rHR).

*Post-transplant metabolic syndrome* (PTMS) was defined as the presence of at least three of the following five conditions as previously described [27]: arterial hypertension (defined as either SBPz and/or DBPz > 1.65 (> 95th pct.) or antihypertensive medication use), obesity (BMIz > 1.65 (> 95th pct.)) and/or central obesity (WCz > 1.28 (> 90th pct.)), increased triglycerides (defined as ≥ 100 mg/dl for 0–9 years of age; ≥ 130 mg/dl for 10–19 years of age; ≥ 150 mg/dl for 20–24 years of age), reduced HDL (defined as < 40 mg/dl), and diagnosis of diabetes mellitus (which included new-onset diabetes after transplantation (NODAT), as well as other types).

*Carotid-femoral pulse wave velocity* (PWV) was assessed using the Vicorder device (Skidmore Medical, Ltd., Bristol, UK) applying the previously described method and reference values [28]. Out of three measurements, the mean was used to calculate *z*-scores for sex and height (PWVz). PWV was considered elevated when PWVz exceeded 1.65 (> 95th pct.).

*Intima media thickness* (IMT) of the carotid artery was measured with a 3–12 MHz linear array transducer (Philips CX50 system, Philips Healthcare, Bothell, USA) in accordance with the Mannheim consensus [29]. One trained operator took five measurements from right and left common carotid artery, about 1–2 cm below its bifurcation. The average of both sides was used to calculate IMT *z*-score for sex and height (IMTz) [30]. IMT was considered elevated when IMTz exceeded 1.65 (> 95th pct.).

*Transthoracic echocardiography* was performed using a Philips CX 50 ultrasound machine (Philips Healthcare, Bothell, USA) equipped with a 1–5 MHz transducer following a standardized protocol in accordance with the guidelines of the American Society of Echocardiography. Left ventricular end diastolic wall thickness and end diastolic dimensions were measured in the parasternal short axis view at the level of the papillary muscles using M-mode. Left ventricular mass index (LVMI) and left ventricular hypertrophy (LVH) (LVMI > 45 g/m^2.16^) were defined as proposed by Chinali et al. [31]. For further assessment of left ventricular diastolic function, we obtained the following parameters from the apical four-chamber view: pulsed wave Doppler measurements of mitral inflow (E, peak early mitral inflow Doppler velocities; A, peak late mitral inflow Doppler velocities; E-wave deceleration time) and peak velocities of early (e´) and late diastolic (a´) excursion of the mitral and septal annulus by tissue Doppler imaging. Five consecutive cardiac cycles were taken from every recording. All parameters were measured five times; the median value was taken for further analysis. To describe diastolic function, E and mitral and septal annular e’ were classified as decreased when < 10th pct., A and E-wave deceleration time as elevated when > 90th pct. in conformance with previously published reference values corrected for body surface area by Cantinotti et al. [32].

### Statistical analysis

Statistical analysis was performed using SAS EG 9.4 (Statistical Analysis Software, Cary, NC, USA). Unless otherwise stated, variables are presented as mean ± standard deviation (SD), categorical variables as percentage along with total number. The association between MVPA and walking-based activity was assessed by Pearson correlation. For univariate statistics, we used an unpaired *t*-test. Using linear regression models, we analyzed the effect of PA on the following CV parameters (endpoints): BP, rHR, PTMS, PWVz, IMTz, LVMI, and echocardiographic diastolic parameters. All models were corrected for age, sex, and eGFR (reflecting graft function). For each endpoint, we calculated two models including either MVPA or MPA and VPA separately. A *p*-value < 0.05 was considered significant.

## Results

### Patient characteristics

A total of 48 patients after pediatric KTx were investigated. Patient characteristics are summarized in Table 1. Mean age was 13.5 ± 4.2 years; 54% (26/48) were male. Mean time since last KTx was 5.5 ± 4.6 years. Of our cohort, 42% (20/48) were transplanted pre-emptively, 21% (10/48) were transplanted from a living-related donor, and 8% (4/48) had already received a second KTx. Those patients in need of dialysis prior to transplantation had a cumulative time on hemo- and/or peritoneal dialysis of 9.3 ± 12.4 months. Mean eGFR at time of investigation was 76.8 ± 37.5 ml/min/1.73 m^2^. The immunosuppressive regimen was mainly based on calcineurin inhibitors.

**Table 1:** Demographic and clinical characteristics of the 48 pediatric KTx recipients

Variables	Mean ± SD	Range	Number (percentage)
Age (years)	13.5 ± 4.2	5–24	
Sex			
Male/female			26/22 (54/46)
Height			
Absolute (cm)	151.3 ± 21.2	109.2–184.0	
*z*-score	0.43 ± 1.31	− 2.88–3.22	
Weight			
Absolute (kg)	49.3 ± 18.4	17.9–96.7	
*z*-score	0.10 ± 1.07	2.51–2.39	
Body mass index			
Absolute (kg/m^2^)	20.7 ± 4.3	14.9–33.4	
*z*-score	0.35 ± 1.03	− 2.28–2.32	
Overweight			8 (17)
Obese			5 (10)
Waist circumference			
Absolute (cm)	74.9 ± 12.7	52.0–108.0	
*z*-score	0.45 ± 0.89	− 1.42–2.06	
Underlying disease			
CAKUT			32 (67)
Non-CACUT			16 (33)
Transplantation			
Preemptive/after prior dialysis			20/28 (42/58)
Re-transplantation			4 (8)
Time since transplantation (years)	5.5 ± 4.6	0–22	
Immunosuppression			
Calcineurin inhibitors			44 (92)
mTOR inhibitors			38 (79)
Mycophenolate mofetil			8 (17)
Steroids			23 (48)
Estimated GFR (ml/min/1.73 m^2^)	76.8 ± 37.5	19.6–200.9	

*CAKUT* congenital anomalies of kidney and urinary tract, *GFR* glomerular filtration rate

### Physical activity

**Fig. 1:**
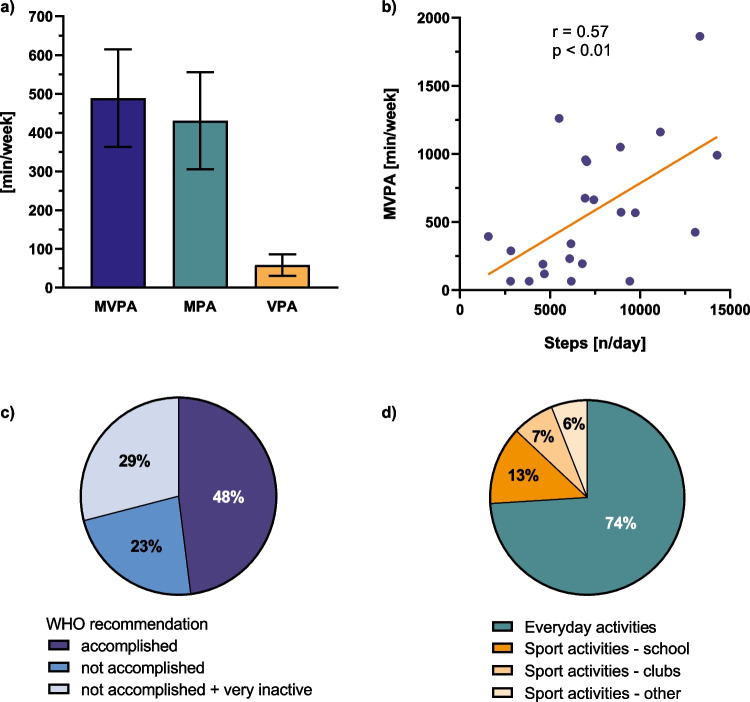
**Physical activity** **of KTx recipients.** **a** Minutes per week of MVPA, MPA, and VPA presented as mean ± standard deviation. **b** Correlation of MVPA (minutes per week, retrieved from MoMo-PAQ [21]) with walking-based activity (number of steps per day, measured by accelerometer) in a subgroup of 23 patients. **c** Percentage of patients accomplishing or not accomplishing the WHO recommendation, i.e., an average of 60 min of PA per day [16]. Note, among those, who did not accomplish the recommended PA amount, there was a subgroup exhibiting very inactive behavior (defined as less than 120 min a week). **d** Percentage of patients engaging in different types of activities. *KTx*, kidney transplantation; *MVPA*, moderate to vigorous physical activity; *MPA*, moderate physical activity; *p*, *p*-value; *PA*, physical activity; *VPA*, vigorous physical activity

The cohort underwent a mean of 489 ± 435 min of MVPA per week, consisting of 431 ± 430 min MPA (88%) and 58 ± 95 min VPA (12%) (Fig. 1a). In a sub-cohort with available accelerometer measurements (*n* = 23), we measured 7317 ± 3406 steps per day, which correlated moderately but significantly with minutes of MVPA (*r* = 0.57, *p* = 0.01; Fig. 1b). The WHO recommendation of an average of 60 min MVPA per day or 420 min per week was fulfilled by 48% (23/48) of the patients, while 52% (25/48) showed an inadequate amount of MVPA. We identified a subgroup of very inactive participants of 29% (14/48), who reported to be active for less than 120 min a week (Fig. 1c).

To further explore the type of activities, we divided MVPA into everyday and sport activities (Fig. 1d). Most of the MVPA was performed as everyday activities (74%) with mainly moderate intensity (MPA 356 ± 421 min vs. VPA 7 ± 37 min). The remainder (26%) was spent on sport activities mainly performed during school (51% sport activities in school, 26% sport activities in sport clubs, 23% other unorganized sport activities). VPA was mostly practiced during sport activities, but 54% (26/48) of our cohort indicated to do no VPA at all.

**Fig. 2:**
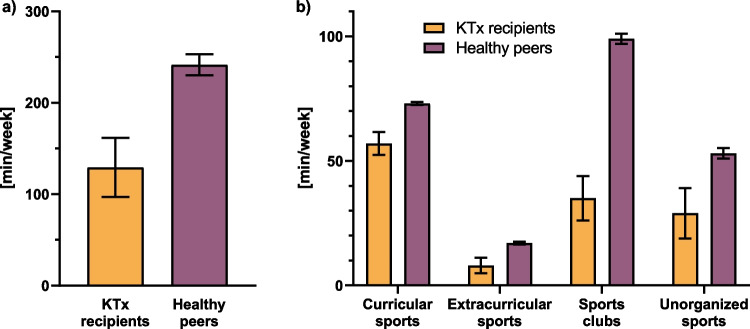
**Comparison of sport activities between KTx recipients and healthy children from the KiGGS cohort** [21]. Minutes per week are shown for **a** the total amount of sport activities presented as mean and 95% confidence interval in whiskers and as **b** stratified by the different sport settings presented as mean with standard error of the mean in whiskers. *KTx*, kidney transplantation

To elucidate how sport activities in pediatric KTx recipients correlated to those performed by healthy peers, we compared our results to available data from the KiGGS cohort (Fig. 2; Table 2). Overall, KTx patients engaged in fewer minutes of sport activities (129; 95%CI, 97–162; Fig. 2a) compared to healthy children and adolescents (242; 95%CI, 230–253; Fig. 2a). This was true also for the different sport settings with the largest discrepancy seen for activities performed in sport clubs (Fig. 2b) and for the comparison of age groups (Table 2). Notably, when looking at the 95%CI, there was no overlap for the total amount of sport between our study population and the KiGGS cohort for all age groups except the age group of 4 to 5 years of age, for which the KTx group consisted of only two patients.

**Table 2:** Sport activities of pediatric KTx recipients compared to children and adolescents from the German Health Interview and Examination Survey (KiGGS, *n* = 3708) [21]

Age	KTx (*n*) vs. KiGGS	Curricular sport (mean ± SD)	Extracurricular sport (mean ± SD)	Sport clubs (mean ± SD)	Unorganized sports (mean ± SD)	Total sporting activities (mean (95%CI))
4–5	KTx (2)	15 ± 21	-	0 ± 0	0 ± 0	15 (− 171–200)
	KiGGS	52 ± 39	-	38 ± 47	24 ± 81	115 (91–125)
6–10	KTx (12)	62 ± 38	10 ± 26	32 ± 45	6 ± 17	110 (55–164)
	KiGGS	80 ± 31	24 ± 38	97 ± 94	35 ± 92	235 (222–248)
11–13	KTx (7)	52 ± 32	16 ± 28	22 ± 29	25 ± 38	115 (19–210)
	KiGGS	87 ± 34	26 ± 55	119 ± 133	57 ± 142	290 (264–316)
14–17	KTx (24)	59 ± 27	5 ± 18	44 ± 73	45 ± 87	153 (103–203)
	KiGGS	65 ± 34	9 ± 32	115 ± 159	82 ± 146	273 (247–299)

Numbers refer to minutes per week. Data from patients above the age of 17 years (*n* = 3) were excluded because of missing comparators from KiGGS

*KTx* kidney transplantation, *SD* standard deviation, *CI* confidence interval

### PA, CV risk factors, and subclinical CV damage

As expected, our study population showed a number of CV risk factors, which are listed in detail in Table 3. Thirty-eight percent (18/48) of patients suffered from PTMS. The most frequent PTMS components were arterial hypertension (85%; 41/48) and hypertriglyceridemia (73%; 36/48). Of the three patients with a known diabetes, two were diagnosed with new-onset diabetes after transplantation (NODAT), one with MODY 5 (*HNF1B* mutation). In addition to the presence of risk factors, a considerable number of patients presented already with CV morbidity. A third (33%; 16/48) presented with elevated PWV, 27% (13/48) with elevated IMT, and 23% (11/47) displayed LVH. Impaired left ventricular relaxation was seen in 26% (12/47) of the patients.

**Table 3:** Cardiovascular parameters

Parameters	Mean ± SD	Range	Number (percentage)
Systolic blood pressure			
Absolute (mmHg)	113.2 ± 10.5	92–145	
*z*-score	0.54 ± 0.84	− 1.17–2.76	
Diastolic blood pressure			
Absolute (mmHg)	67.0 ± 10.3	50–104	
*z*-score	0.39 ± 0.98	− 1.04–3.93	
Resting heart rate (bpm)	84.4 ± 14.0	55.5–117.5	
Blood lipids			
Triglycerides (mg/dl)	222.9 ± 169.2	64.0–1021.0	
Total cholesterol (mg/dl)	200.0 ± 57.2	108.0–164.0	
HDL cholesterol (mg/dl)	52.3 ± 13.7	31.0–78.0	
LDL cholesterol (mg/dl)	123.7 ± 39.9	51.0–250.0	
Post-transplant metabolic syndrome			18 (38)
Arterial hypertension			41 (85)
Obesity and/or central obesity			8 (17)
Diabetes			3 (6)
Increased triglycerides			36 (73)
Reduced HDL			12 (25)
Pulse wave velocity			
Absolute (m/s)	5.6 ± 0.7	4.4–6.8	
*z*-score	1.15 ± 1.12	− 1.11–3.84	
Elevated			16 (33)
Intima media thickness			
Absolute (mm)	0.43 ± 0.04	0.34–0.52	
*z*-score	1.15 ± 0.97	− 0.65–3.56	
Elevated			13 (27)
Left ventricular mass (g)	102.2 ± 48.4	30.2–268.9	
Left ventricular mass index (g/m^2.16^)	38.3 ± 11.0	23.2–84.5	
Left ventricular hypertrophy			11 (23)
Diastolic function			
E velocity (cm/s)	105.4 ± 16.7	68.4–134.0	
A velocity (cm/s)	60.4 ± 13.5	38.4–91.1	
E/A ratio	1.8 ± 0.5	1.0–3.0	
E-wave deceleration time (ms)	164.4 ± 30.5	111.0–267.5	
Mitral annular e’ (cm/s)	17.5 ± 2.8	12.8–24.3	
Mitral annular e/e’	6.2 ± 1.5	3.2–9.8	
Septal annular e’ (cm/s)	12.7 ± 2.2	8.2–19.1	
Septal annular e/e’	8.5 ± 1.8	5.4–13.4	
Impaired left ventricular relaxation			12 (25)

*HDL* high density lipoprotein, *LDL* low density lipoprotein, *E* peak early mitral inflow Doppler velocity, *A* peak late mitral inflow Doppler velocity, *e’* early diastolic annular myocardial velocity

The comparison of pediatric KTx recipients with adequate MVPA levels with those showing an inadequate amount revealed a number of differences (Suppl. Table 1). Most importantly, we found significantly higher eGFR in the group with adequate MVPA. We therefore performed linear regression models adjusted for age, sex, and graft function (eGFR) to further describe the effect of PA on the different CV parameters.

#### CV risk factors

**Fig. 3:**
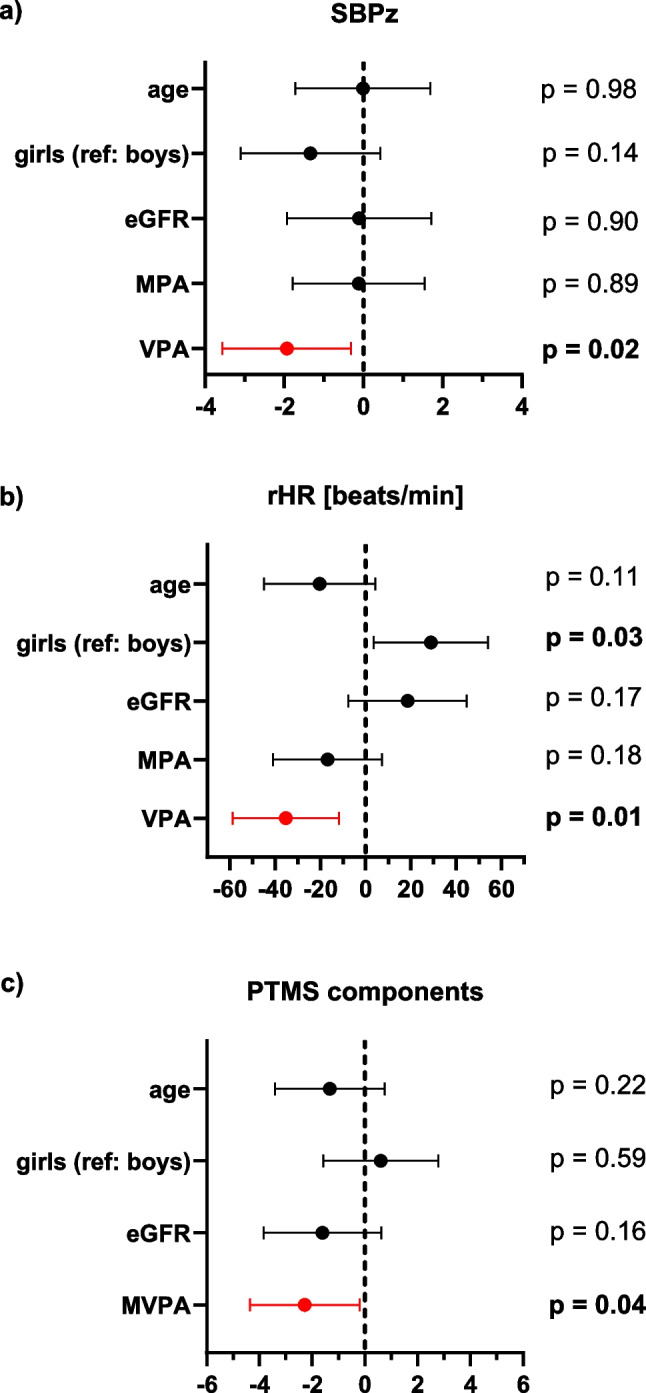
**Beneficial effects of PA on different CV parameters**. **a** SBPz, **b** rHR, and **c** number of PTMS components. Forest plots depict the standardized ß and 95% confidence interval derived from the linear regression models for the respective endpoint. All models were adjusted for sex, age, and graft function (eGFR). eGFR, glomerular filtration rate; MPA, moderate intensity physical activity; PTMS, components of post-transplant metabolic syndrome, rHR, resting heart rate; SBPz, systolic blood pressure *z*-score; VPA, vigorous intensity physical activity; MVPA, moderate to vigorous intensity physical activity

Multivariable analysis revealed that VPA was a significant predictor of a lower SBPz and a lower rHR (Fig. 3a, b). It showed that 60 min more of VPA per week was associated with a decrease of SBPz by 0.18 and rHR by 3 beats per minute. The amount of MVPA or MPA had no significant effect on either SBPz or rHR. No relationship of PA with DBPz was seen for any of the parameters. When analyzing the endpoint PTMS, we found that increasing amounts of MVPA were associated with having fewer PTMS components (Fig. 3c). An additional 22 h per week of MVPA was associated with one component less of PTMS. This amount of activity corresponds to the difference between the most and the least active patients in our cohort.

#### Subclinical CV damage

**Fig. 4:**
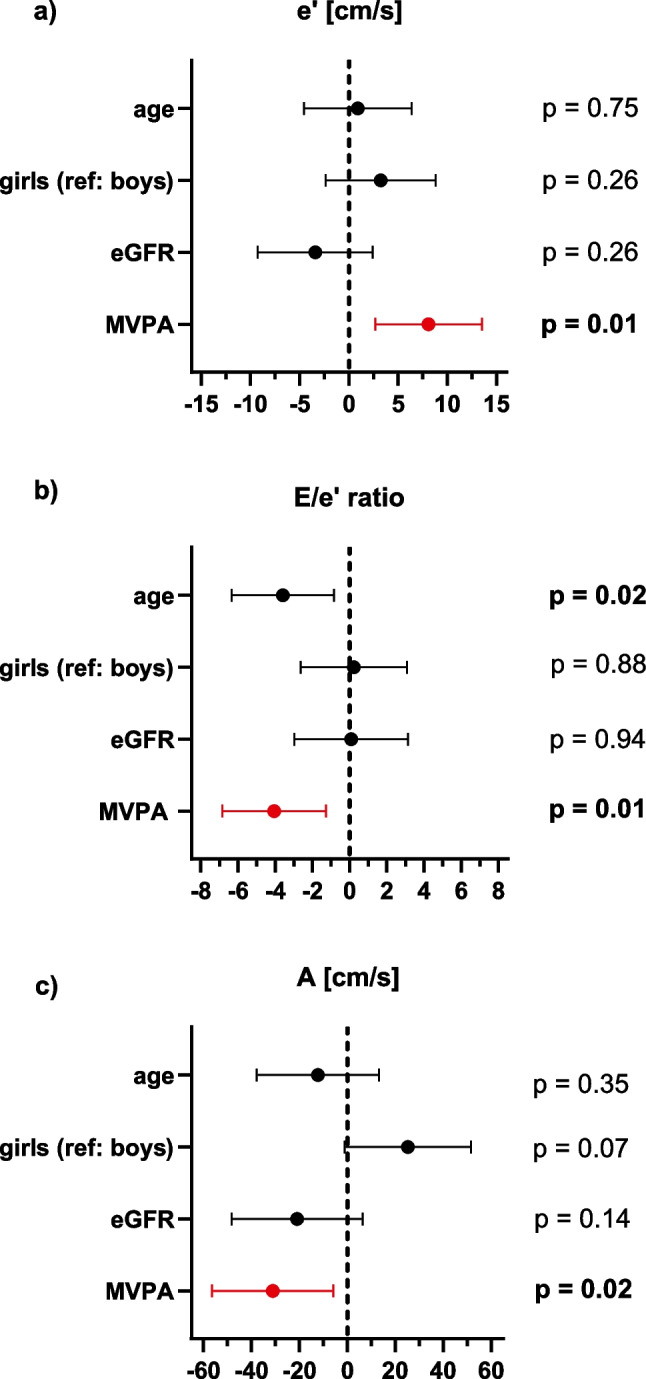
**Beneficial effects of PA on left ventricular diastolic function**. **a** mitral annular e’ velocity, **b** mitral annular E/e’ ratio, and **c** mitral A velocity. Forest plots depict the standardized ß and 95% confidence interval derived from the linear regression models for the respective endpoint. All models were adjusted for sex, age, and graft function (eGFR). eGFR, glomerular filtration rate; MPA, moderate intensity physical activity; VPA, vigorous intensity physical activity; MVPA, moderate to vigorous intensity physical activity; e´, peak velocities of early excursion by tissue Doppler imaging; E, peak early mitral inflow Doppler velocities; A, peak late mitral inflow Doppler velocities

We saw no effect of PA on the vascular endpoints PWVz or IMTz. While we could also not find an effect of PA on LVMI, the multivariable analysis showed a beneficial effect of MVPA on mitral annular e’ and E/e´ ratio (Fig. 4a, b). More MVPA was significantly associated with a higher mitral annular e’ and lower E/e´ ratio, indicating better left ventricular compliance and less left ventricular filling pressures. Consistent with these findings, multivariable analysis revealed a significant correlation of MVPA and mitral A velocity (indicating a larger contribution of atrial contraction to left ventricular filling). With increasing MVPA the A-wave decreased significantly in our cohort (Fig. 4c). We saw no effect of PA on E, E-wave deceleration time, or E/A ratio.

## Discussion

Our study demonstrates that young KTx recipients were considerably less active than their healthy peers. This difference was especially pronounced when looking at sport activities, in which KTx patients rarely engaged, especially in sport outside of the school’s curriculum. Along with this finding, we saw a specific lack of activities with vigorous intensity. Notably, we identified one third of our KTx recipients with an extremely sedentary lifestyle. In our cohort, we were able to show that PA had positive effects on CV parameters. VPA reduced SPB and rHR, while MVPA was associated with fewer PTMS components and better LV diastolic function.

While previous studies have indicated low levels of PA in children and adolescents after KTx [33, 34], our study extends these findings by demonstrating that KTx recipients display a very different pattern of their activities and particularly lack PA of vigorous intensity. The greatest amount of MVPA was reached through everyday activities of moderate intensity. The comparison with healthy peers from the KiGGS study revealed that KTx recipients participated considerably less in sport activities. VPA usually practiced during sport is a major cornerstone of the WHO guideline, which strongly recommends VPA for at least three times a week to gain most health benefits. It is therefore of particular importance that half of our study cohort did not engage in VPA at all and the remainder barely reached the WHO goal. Furthermore, we identified an alarming proportion of approximately one third (29%) of the study population, who indicated an extremely inactive lifestyle with less than 120 min of MVPA per week. For comparison, only 9% of healthy children and adolescents report a comparable low level of PA [35]. Reasons for less PA participation among children and adolescents with CKD were reported by Wolf and colleagues, who found that fatigue and illness seemed the main reasons before transplantation, after pediatric KTx the prevailing concern was injury to the transplant [33]. Such concerns could be easily overcome through an adequate education on sport activities that are ideal for this group of patients and speaks for an interdisciplinary approach with the involvement of specialists from sports medicine in the post-transplant care.

The lack of VPA seen in our cohort becomes even more important in light of its impact on SBPz and rHR. Recent literature in healthy children and adolescents suggests that especially PA with higher intensity results in an improved CV risk profile with lower BMI, better aerobic fitness (VO_2_ max) [36] and lower systolic BP [37]. Further support comes from Hay et al. who also showed an association of VPA with lower systolic BP [38]. These studies only comprise healthy children and adolescents. Our data suggesting similar CV health benefits after KTx is new and of particular importance as optimal BP control is crucial for the prevention of CV target organ damage and protection of graft function in pediatric KTx recipients [2, 39]. As BP control is often unsatisfactory in these patients despite the combination of several antihypertensive drugs [40], our findings promote lifestyle modification with the implementation of sport activities and particularly VPA as an important cornerstone of post-transplant care. Importantly, the prevalence of overweight and obesity was low in our cohort when compared to other KTx cohorts [41]. The fact that we did not find an association of PA on BMI does not mean that pediatric KTx recipients in general will not profit from PA with regard to their weight. This also applies to diabetes, which is an important complication after transplantation. In our cohort, the number of patients affected was small, and the underlying pathomechanisms responsible for manifestation were heterogeneous. Despite these differences, dysglycemia is expected to be associated with higher CV morbidity and mortality. While a recent study in adult KTx recipients showed that exercise training improved glucose metabolism [42], an association of PA on glucose metabolism could not be analyzed in our study due to small sample size.

But not only was VPA positively associated with parameters of CV health: an increase in the combination of moderate and vigorous activities (reflected by minutes of MVPA) was sufficient to affect PTMS components in our cohort of young KTx recipients. PTMS has been shown as a trigger for CV damage in adult KTx recipients. A high prevalence of PTMS in transplanted children and adolescents has been reported [6] showing a 16-fold higher risk for PTMS in KTx recipients compared to healthy peers. Findings in healthy children and adolescents [43] and a few studies in transplanted pediatric patients suggest a beneficial effect of PA on the prevalence of PTMS. Tangeraas et al. investigated a small cohort of 22 children and adolescents after KTx and showed that those with none or one metabolic risk factor were more active than those with a greater number of risk factors [44]. Blöte et al. demonstrated that PA decreased the risk of PTMS in pediatric solid organ and stem cell recipients [6]. Our study extends these findings by providing more detail on PA such as durations, intensities, and settings. Knowing that 3 more hours per day of MVPA, which means, e.g., playing soccer in the garden or cycling to school, might reduce the prevalence of PTMS in these patients is very helpful in daily practice.

Despite significant structural vascular changes present in our cohort, we did not see an effect of PA on PWV or IMT. While recent literature revealed cardiorespiratory fitness as preventive for small and large vessel disease in children at risk [45], the effect of PA on vascular alterations in children after KTx remains understudied. In adults, an exercise intervention significantly reduced PWV in a cohort of 42 KTx recipients [46]. More PA and greater cardiorespiratory fitness were associated with lower IMT in glucose-intolerant adult KTx recipients [47]. In healthy children, especially the time spent on VPA was associated with lower PWV [48]. We cannot confirm these findings for our cohort and can only speculate on the reasons. One explanation could be the high burden of vascular morbidity in our cohort that was not modifiable by the amount of PA performed in our cohort; another explanation refers to a potential publication bias towards studies showing no effect of PA on vascular phenotypes. Only a longitudinal study design, ideally with an interventional exercise approach, can address the remaining question of how PA affects vascular target organ damage in this very high-risk group of patients.

While we did not see an effect on LVMI, our data indicates a beneficial effect of MVPA on LV diastolic function suggesting an overall improved cardiac phenotype in KTx recipients engaging in PA. A previous study showed impaired diastolic function in children with CKD that persisted following KTx [7]. In our cohort, a higher mitral annular e’ and lower E/e´ ratio reflecting a better LV compliance were associated with increasing time spent on MVPA. As findings from a cardiac MRI study in adult hemodialysis patients pointed out that the interventricular septum is particularly prone to the development of myocardial fibrosis [49], the positive effect on the mitral annulus seen in our cohort might therefore be of even greater importance.

Our study has certain limitations. It has been suggested that the use of questionnaires may overestimate PA as compared to other approaches [50]. Similar to our own results, others have demonstrated a significant correlation between MVPA measured by the MoMo-PAQ and accelerometer measurements [22]. While there may be a remaining risk that we somewhat overestimated the real amount of PA in our study participants, the great advantage of using the questionnaire was the possibility to compare our cohort with a representative cohort of healthy children and adolescents. Furthermore, the assessment of PA intensities using children-specific MET values allowed exploring the impact of intensity levels on the different endpoints. Our medium-sized cohort may have been too small to describe all expected associations, especially in a cross-sectional setting. However, we were able to show some meaningful associations in this very well-phenotyped cohort that extend our current knowledge. Ideally, longitudinal assessments, larger sample sizes, and interventional studies in a multicenter approach are needed to further elucidate the influence of PA on CV parameters and CV target organ damage in pediatric KTx recipients.

## Conclusion

Our study demonstrated that there is a considerable lack of PA in children and adolescents after KTx, especially with regard to activities of higher intensity. Importantly, even in this medium-sized cohort, PA was associated with a significant beneficial impact on CV health. Thus, CV prevention strategies in young KTx recipients should also address PA behavior and must include adequate education to debunk reservations about sport activities in patients and their families. Further studies must elucidate which sport activities, type, frequency, and duration wise, are best suited for this group of patients. This must take the safety of the graft into account. Long-term CV protection including lifestyle changes through PA could be promising in pediatric KTx recipients and should therefore be further investigated in clinical trials.

### Supplementary Information

Below is the link to the electronic supplementary material.

Graphical abstract (PPTX 191 KB)

Supplementary file2 (DOCX 20 KB)

## Data Availability

Data available upon reasonable request to the authors.
