# Supplementary material for: Physical activity and its impact on cardiovascular health in pediatric kidney transplant recipients
Source: Pediatr Nephrol. 2023 Dec 16;39(5):1587–98. doi: 10.1007/s00467-023-06248-7 (PMC10943152; doi:10.1007/s00467-023-06248-7)
Supplement: Supplementary file 1 — Graphical abstract (PPTX 191 KB) [file 467_2023_6248_MOESM1_ESM.pptx]

## Slide 1
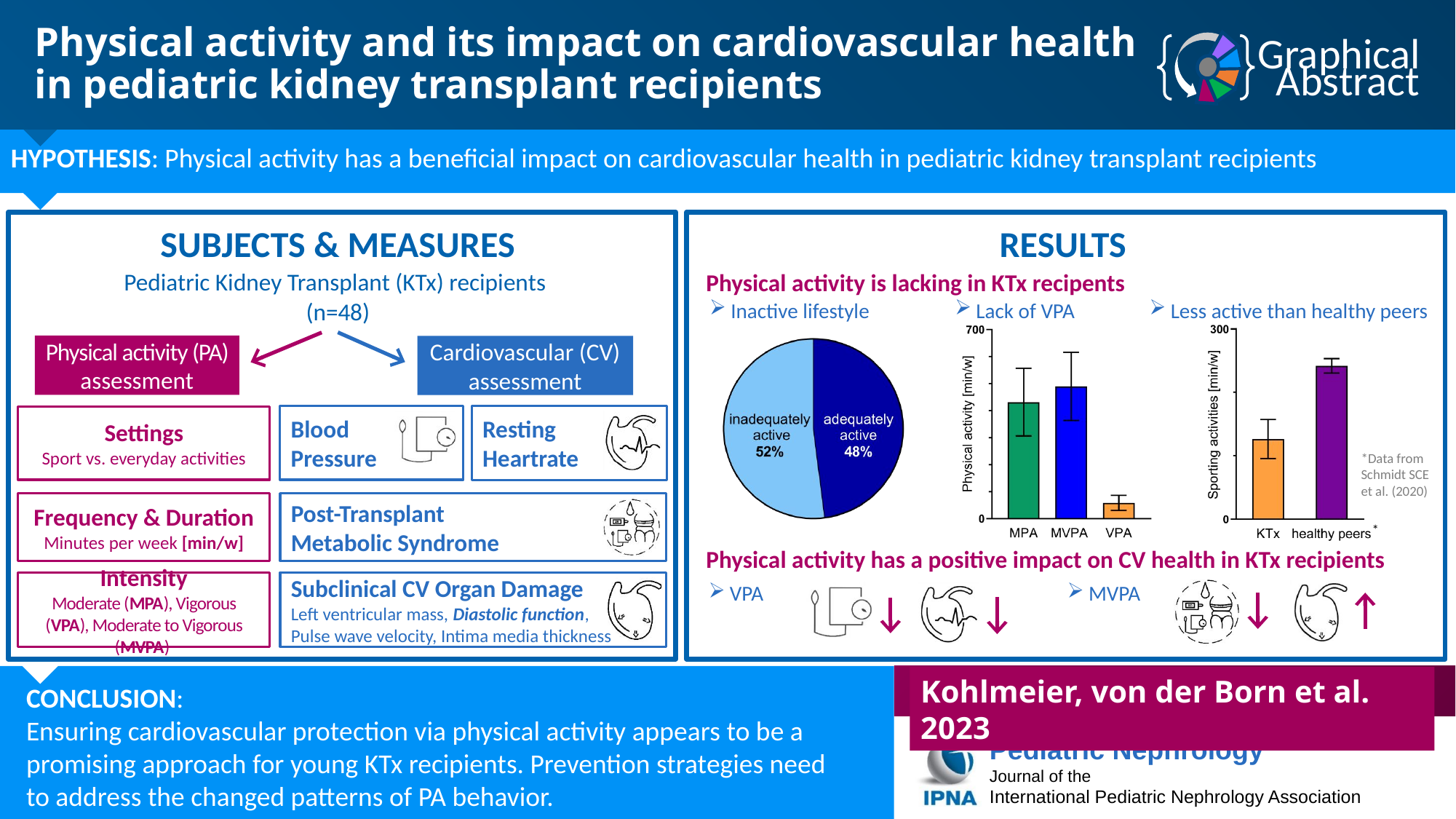

Physical activity and its impact on cardiovascular health
in pediatric kidney transplant recipients
HYPOTHESIS: Physical activity has a beneficial impact on cardiovascular health in pediatric kidney transplant recipients
Subjects & Measures
Pediatric Kidney Transplant (KTx) recipients
(n=48)
Physical activity (PA) assessment
Cardiovascular (CV) assessment
Blood
Pressure
Resting Heartrate
Settings
Sport vs. everyday activities
Post-Transplant
Metabolic Syndrome
Frequency & Duration
Minutes per week [min/w]
Subclinical CV Organ Damage
Left ventricular mass, Diastolic function,
Pulse wave velocity, Intima media thickness
Intensity
Moderate (MPA), Vigorous (VPA), Moderate to Vigorous (MVPA)
Results
Physical activity is lacking in KTx recipents
Lack of VPA
Less active than healthy peers
Inactive lifestyle
*Data from Schmidt SCE et al. (2020)
*
Physical activity has a positive impact on CV health in KTx recipients
VPA
MVPA
Kohlmeier, von der Born et al. 2023
CONCLUSION:
Ensuring cardiovascular protection via physical activity appears to be a promising approach for young KTx recipients. Prevention strategies need to address the changed patterns of PA behavior.
