# Supplementary material for: Physical activity and its impact on cardiovascular health in pediatric kidney transplant recipients
Source: Pediatr Nephrol. 2023 Dec 16;39(5):1587–98. doi: 10.1007/s00467-023-06248-7 (PMC10943152; doi:10.1007/s00467-023-06248-7)
Supplement: Supplementary file 2 — Supplementary file2 (DOCX 20 KB) [file 467_2023_6248_MOESM2_ESM.docx]

**Supplemental Material**

**Physical activity and its impact on cardiovascular health in pediatric kidney transplant recipients**

Lena Kohlmeier^1^*; Jeannine von der Born MD^1^*; Elena Bauer MSc^1^; Kerstin Fröde MD^1^; Carl Grabitz MD^1^; Anne-Sophie Greiner^1^; Alexander A. Albrecht MD^2^; Nima Memaran MD^1^; Rizky I. Sugianto DDS, PhD^1^; Uwe Tegtbur MD^2^; Bernhard MW Schmidt MD, MSc^3^; Nele Kanzelmeyer MD^1^; Anette Melk MD, PhD^1^

^1^Department of Pediatric Kidney, Liver and Metabolic Diseases, Hannover Medical School, Hannover,

Germany

^2^Institute of Sports Medicine, Hannover Medical School, Hannover, Germany

^3^Department of Nephrology and Hypertension, Hannover Medical School, Hannover, Germany

* L. Kohlmeier and J. von der Born contributed equally

**Corresponding author:** Anette Melk, MD, PhD

Department of Pediatric Kidney, Liver and Metabolic Diseases; Hannover Medical School; Carl-Neuberg-Str. 1; 30625 Hannover; Germany

Email: [melk.anette@mh-hannover.de](mailto:melk.anette@mh-hannover.de)

<https://twitter.com/AnetteMelk>

ORCID: 0000-0002-8164-6318

| **Supplemental Table 1**:  Comparison of pediatric KTx recipients with an inadequate level of moderate to vigorous intensity physical activity (MVPA: <420 minutes per week) and pediatric KTx recipients with adequate MVPA levels (MVPA: ≥420 minutes per week). | | | | | |
| --- | --- | --- | --- | --- | --- |
|  | **Inadequately active   (n=25)** | | **Adequately active  (n=23)** | | **p-value*** |
|  | **Mean** | **SD** | **Mean** | **SD** |  |
| Age [years] | 14.1 | 3.7 | 12.8 | 4.7 | 0.29 |
| Height z-score | -0.54 | 1.29 | -0.31 | 1.34 | 0.54 |
| Weight z-score | -0.04 | 1.14 | 0.26 | 1.00 | 0.33 |
| BMIz | 0.22 | 1.10 | 0.49 | 0.95 | 0.36 |
| WCz | 0.47 | 0.94 | 0.42 | 0.84 | 0.84 |
| Time since KTx [years] | 6.5 | 5.2 | 5.2 | 4.8 | 0.39 |
| **eGFR** **[ml/min/1.73m^2^]** | **66.4** | **28.4** | **88.1** | **43.2** | **0.04** |
| Resting heart rate [bpm] | 85.1 | 14.4 | 83.7 | 13.9 | 0.73 |
| SBPz | 0.61 | 0.86 | 0.45 | 0.82 | 0.51 |
| DBPz | 0.14 | 0.74 | 0.59 | 1.16 | 0.11 |
| **Antihypert. medication [number]** | **2.6** | **1.8** | **1.5** | **1.2** | **0.02** |
| Triglycerides [mg/dl] | 247.4 | 201.0 | 197.4 | 127.7 | 0.31 |
| Total cholesterol [mg/dl] | 221.6 | 62.4 | 201.0 | 49.0 | 0.52 |
| HDL [mg/dl] | 49.0 | 13.2 | 55.8 | 13.6 | 0.09 |
| LDL [mg/dl] | 130.9 | 41.3 | 116.1 | 37.8 | 0.21 |
| **PTMS components [number]** | **2.4** | **1.0** | **1.7** | **1.0** | **0.01** |
| PWVz | 0.87 | 1.03 | 1.44 | 1.17 | 0.10 |
| IMTz | 1.23 | 1.05 | 1.07 | 0.90 | 0.56 |
| LVMI [g/m^2.16^] | 40.5 | 13.3 | 35.9 | 7.6 | 0.16 |
| E velocity [cm/s] | 106.4 | 16.1 | 104.4 | 17.7 | 0.69 |
| **A velocity [cm/s]** | **64.6** | **14.2** | **56.1** | **11.5** | **0.03** |
| E/A ratio | 1.7 | 0.4 | 1.9 | 1.7 | 0.12 |
| Mitral annular E/e’ ratio | 6.4 | 1.6 | 6.0 | 1.4 | 0.39 |
| Septal annular E/e’ ratio | 8.6 | 1.8 | 8.3 | 1.8 | 0.57 |
| **Steps per day [number]** | **5001** (n=11) | **2226** | **9440** (n=12) | **2901** | **<0.01** |
| Definition based on the WHO physical activity guideline [16] MVPA, moderate to vigorous physical activity; BMIz, z-score of Body mass index; WCz, z-score of waist circumference; SBPz, z-score of systolic blood pressure; DBPz, z-score of diastolic blood pressure; PWVz, z-score of carotid-femoral Pulse wave velocity; IMTz, z-score of carotid intima media thickness  *unpaired t-test | | | | | |
